# Correction: Faught et al. “Socioeconomic Disadvantage across the Life Course is associated with Diet Quality in Young Adulthood” *Nutrients*, 2019, 11(2), 242

**DOI:** 10.3390/nu11081807

**Published:** 2019-08-05

**Authors:** Erin L. Faught, Lindsay McLaren, Sharon I. Kirkpatrick, David Hammond, Leia M. Minaker, Kim D. Raine, Dana Lee Olstad

**Affiliations:** 1 Department of Community Health Sciences, Cumming School of Medicine, University of Calgary, Teaching, Research, and Wellness Building, 3280 Hospital Drive NW, Calgary, AB T2N 4Z6, Canada; 2 School of Public Health and Health Systems, University of Waterloo, 200 University Avenue West, Waterloo, ON N2L 3G1, Canada; 3 School of Planning, University of Waterloo, 200 University Ave, Waterloo, ON N2L 3G1, Canada; 4 School of Public Health, University of Alberta, University of Alberta, 4-077 Edmonton Clinic Health Academy, 11405-87 Ave, Edmonton, AB T6G 1C9, Canada

The authors wish to make a correction to the published version of their paper [1]. 

We noticed an error in the text that requires correcting as it may contribute to an incorrect understanding of our study’s scientific conclusions. In the text accompanying Table 4 (Section 3.3: Mediation Analysis (Pathways Hypothesis), page 11), the third sentence in the paragraph reads: “Adult SEP at the level of university education mediated associations between childhood SEP and mean adult HEI-2015 score (*p* < 0.001).” This should in fact read: “Adult SEP at the level of high school or less mediated associations between childhood SEP and mean adult HEI-2015 score (*p* < 0.001).” This correction is consistent with the results presented in Table 4, and in line with the scientific conclusions discussed in the article that a low socioeconomic position (SEP) in adulthood mediates associations between childhood SEP and adult diet quality. SEP at the level of university education was the reference group in this analysis. 

In addition, we would like to clarify that multinomial regression was used for the analysis pertaining to the results presented in the first row of Table 4, which examine the associations between childhood SEP and adult SEP.

The authors apologize to the readers for any inconvenience caused by this modification. This change does not impact the text of the paper, the overall results, or the scientific conclusions. The original manuscript will remain online on the article webpage with a reference to this correction.
